# Experimental Method for the Measurements and Numerical Investigations of Force Generated on the Rotating Cylinder under Water Flow

**DOI:** 10.3390/s21062216

**Published:** 2021-03-22

**Authors:** Teresa Abramowicz-Gerigk, Zbigniew Burciu, Jacek Jachowski, Oskar Kreft, Dawid Majewski, Barbara Stachurska, Wojciech Sulisz, Piotr Szmytkiewicz

**Affiliations:** 1 Faculty of Navigation, Gdynia Maritime University, 81-225 Gdynia, Poland; z.burciu@wn.umg.edu.pl (Z.B.); j.jachowski@wn.umg.edu.pl (J.J.); 2 AREX Ltd., 81-212 Gdynia, Poland; oskar.kreft@arex.pl; 3 Institute of Hydro-Engineering of Polish Academy of Sciences, 80-328 Gdansk, Poland; d.majewski@ibwpan.gda.pl (D.M.); barbarastachurska@ibwpan.gda.pl (B.S.); wojciechsulisz@ibwpan.gda.pl (W.S.); piotrszmytkiewicz@ibwpan.gda.pl (P.S.)

**Keywords:** rotating cylinder, force sensor with built-in amplifier, strain gauge sensor, CFD analysis

## Abstract

The paper presents the experimental test setup and measurement method of hydrodynamic force generated on the rotating cylinder (rotor) under uniform flow including the free surface effect. The experimental test setup was a unique construction installed in the flume tank equipped with advanced flow generating and measuring systems. The test setup consisted of a bearing mounted platform with rotor drive and sensors measuring the hydrodynamic force. The low length to diameter ratio cylinders were selected as models of bow rotor rudders of a shallow draft river barge. The rotor dynamics was tested for the rotational speeds up to 550 rpm and water current velocity up to 0.85 m/s. The low aspect ratio of the cylinder and free surface effect had significant impacts on the phenomena influencing the generated hydrodynamic force. The effects of the rotor length to diameter ratio, rotational velocity to flow velocity ratio, and the Reynolds number on the lift force were analyzed. The validation of the computational model against experimental results is presented. The results show a similar trend of results for the simulation and experiment.

## 1. Introduction

The list of variables used in the paper is presented in Table 1.

The field experiments [1] of a self-propelled remotely operated river push barge model in 1:20 geometrical scale equipped with the bow steering system consisting of two rotating cylinders (rotors) showed the significant enhancement of her motion control due to the rotor-generated hydrodynamic force.

**Figure 1:**
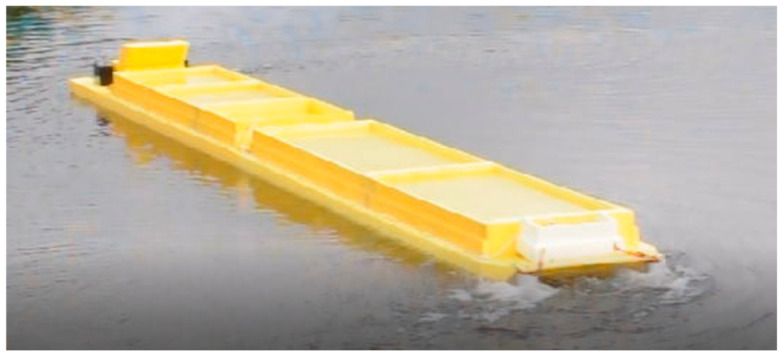
The push barge model in 1:20 geometrical scale during field experiments.

The push barge model during the field experiments is presented in Figure 1.

The model tests followed by the numerical simulation confirmed that the hydrodynamic steering force generated by the bow system was dependent on rotors dimensions, their rotational speed, water inflow velocity, and shape of the barge bow [2,3].

The future application of the bow steering system for autonomous river barges is related to the development of an algorithm for the rotor angular velocity control based on the reliable model of the generated hydrodynamic force. The implementation of this algorithm in the ship-handling model along with the automation of main on-board systems and their control integration in a single communication channel is the first step of transition from level zero to level one of autonomy, e.g., remote vessel controlled by a human operator [4].

The program of the tests presented in the paper is adapted to the rotor models in a larger geometrical scale than the scale used in the field experiments, e.g., 1:10. The velocity scale according to the Froude principle of similarity is $1:\sqrt{10}$. The assumed rotational velocity is in the range of 0–550 rpm and the Reynolds number related to the flow is in the range 2 × 10^4^–1.23 × 10^5^ The examined cylinder aspect ratios are chosen as 0.5, 1, and 1.5. The round endplates are installed on the cylinder to reduce the strength of the expected tip vortices affecting the hydrodynamic force [5].

This paper presents a method developed for the measurements of the hydrodynamic force generated on a rotating cylinder under the water current and the experimental test setup constructed for the model tests. The verification and validation methods of the hydrodynamic force computed using the CFD (Computation Fluid Dynamics) FLOW-3D program are proposed.

The experimental test setup presented in the paper is a unique construction consisting of a bearing mounted platform with the rotor drive and sensors measuring the components of the hydrodynamic force, installed in the flume tank equipped with an advanced wave generator and system for measurements of the flow field parameters.

The available experimental results for the prediction of the rotor-generated flow field are mainly based on the experiments carried out in wind tunnels or carried out in the water without the free surface effect. The published experimental and numerical data related to both the rotor aerodynamics and hydrodynamics present a different combination of the significant influencing parameters.

The endplate diameter to cylinder diameter ratio D_e_/D and the cylinder aspect ratio (length to diameter ratio) L/D were studied by Thouault et al. [5]. Their general conclusions from CFD simulations are in line with the results presented in this study. The flow separates on each endplate edge and the impact of tip vortices on the flow field generation is significant at high rotational rates, especially for low-aspect-ratio cylinders with small endplates. The obtained lift coefficient (C_L_) was equal to 4.71 for the cylinder with L/D = 1.3, D_e_/D = 2, α = 2, and Re = 7.1 × 10^4^. The free surface effect was not included in that study.

The unsteady flow past a rotating circular cylinder at flow related Reynolds numbers Re = 1 × 10^3^ and Re = 1 × 10^4^, and cylinder rotation rate ratio α (tangential velocity to free steam velocity ratio) between 0.5 and 3, was investigated both theoretically and experimentally by Badr et al. [6]. This experimental investigation based on flow visualization showed the difference between numerical and experimental results especially at the highest rotation rates, when the three-dimensional effects become significant.

Karabelas et al. [7] presented lift, drag, and hydrodynamic moment coefficients obtained from numerical modeling at Re = 5 × 10^5^, Re = 1 × 10^6^, and Re = 5 × 10^6^.

Chen and Rheem [8] presented the experiments carried out for different aspect ratios of cylinders for Re = 1 × 10^5^ and α in the range between 0 and 8.

Zhou et al. (2015) [9] presented the experimental study on the water flow past a non-rotating circular cylinder at Re = 7 × 10^3^–8 × 10^4^, considering the different groove patterns on the cylinder surface.

Tokumaru & Dimotakis [10] described the influence of forced rotary oscillations added to the steady rotation on the wake separation and the mean lift force for the cylinder of the large aspect ratio equal to 18.7. They presented the lift coefficient values obtained from measurements and compared with other published results at Re = 3.8 × 10^3^–1.2 × 10^5^ and cylinder aspect ratios 4.7–18.8. For α = 1, the lift coefficient values were between 0.8 and 1.2, and for α = 2 they were between 3 and 4, which can be compared with the results of measurements presented in this paper for the cylinder with aspect ratio 1.5 when the free surface effect at small rotational speed can be neglected.

Wong et al. [11] presented the results of experimental investigations of flow-induced rotor vibrations at flow dependent Reynolds numbers in the range of 1.1 × 10^3^–6.3 × 10^3^ and noticed the vortex shedding cease and decrease of the oscillation amplitude at α = 2. The observed phenomena were significantly different from the comparative CFD predictions and other experimental results at lower Reynolds numbers described by Bourguet & Jacono [12].

Carstensen et al. [13] presented the experimental test setup for the measurements of the hydrodynamic force on the rotating spar buoy model. The results included lift and drag coefficients at Re in the range of 3.2 × 10^4^ to 9.9 × 10^4^ and α between 0–4. In these experiments, the tested cylinder was divided into three parts. Only the middle part with diameter 0.160 m and aspect ratio 2.5 was rotating. The free surface effect was limited due to the not rotating upper part of the cylinder. The bottom tip vortices were limited by not rotating the lower part of the cylinder. The results included lift force coefficients at Re in the range of 1 × 10^4^ to 6 × 10^4^ and α from 0 to 5. The lift coefficient for α = 2 was equal to 4.

In the experiment presented by Wang et al. [14], the tested cylinder was installed under the plate situated on the water surface, eliminating the free surface effect and tip vortices on the top of the cylinder. For the cylinder of 0.1 m diameter and aspect ratio 1, at Reynolds numbers in the range of from Re = 2 × 10^5^ to 1 × 10^6^ and α = 2, the lift coefficient was equal to 3.5.

Mobini & Niazi [15] presented the lift coefficient at different Reynolds numbers computed using the Large Eddy Simulation method. For Re = 6 × 10^4^ and α = 1, C_L_ was equal to 0.75, compared to C_L_ = 1.15 presented by Karabelas [7]. For Re = 1 × 10^4^ and α = 1.5, C_L_ was equal to 1.95, and for α = 2, it was equal to 2.75.

The research presented in this paper demonstrates the sensors used in the experiment and accuracy of measurements determined by comparing the obtained results with available published data. CFD simulation of rotor generated hydrodynamic force was carried out and validated for the same ranges of parameters as the experiment.

## 2. Materials and Methods

### 2.1. Hydrodynamic Lift Force Measurements

**Figure 2:**
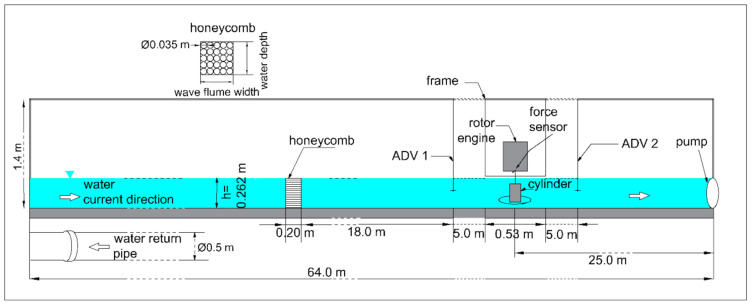
Scheme of the measurement area.

The measurements presented in the paper were carried out in the wave flume of the hydraulic laboratory of the Institute of Hydro-Engineering of Polish Academy of Sciences in Gdansk. The wave flume was 64.0 m long, 0.6 m wide, and 1.40 m high with side walls made of 0.018 m thick glass sheets allowing for visualization of the measurements. The scheme of the measurement area is presented in Figure 2.

The flume is equipped with the FLYGT pp4640 power turbines, which, depending on the water depth, can generate water flows with velocity from 0.1 m/s to 1.0 m/s in both directions [16,17]. The water current generation is electronically controlled and ensures the stability of the mean flow velocity with deviations no greater than 2%. The flow is regulated by the honeycomb, which is a flow straightening structure used to avoid large eddies that can be generated by pump and to minimize the turbulence effects of the flow [18]. The honeycomb is composed of pipes of 0.2 m length and 0.035 m diameter each, vertically positioned along the flow axis. The water circulation is provided by return pipe with the diameter of 0.5 m located under the wave flume.

**Figure 3:**
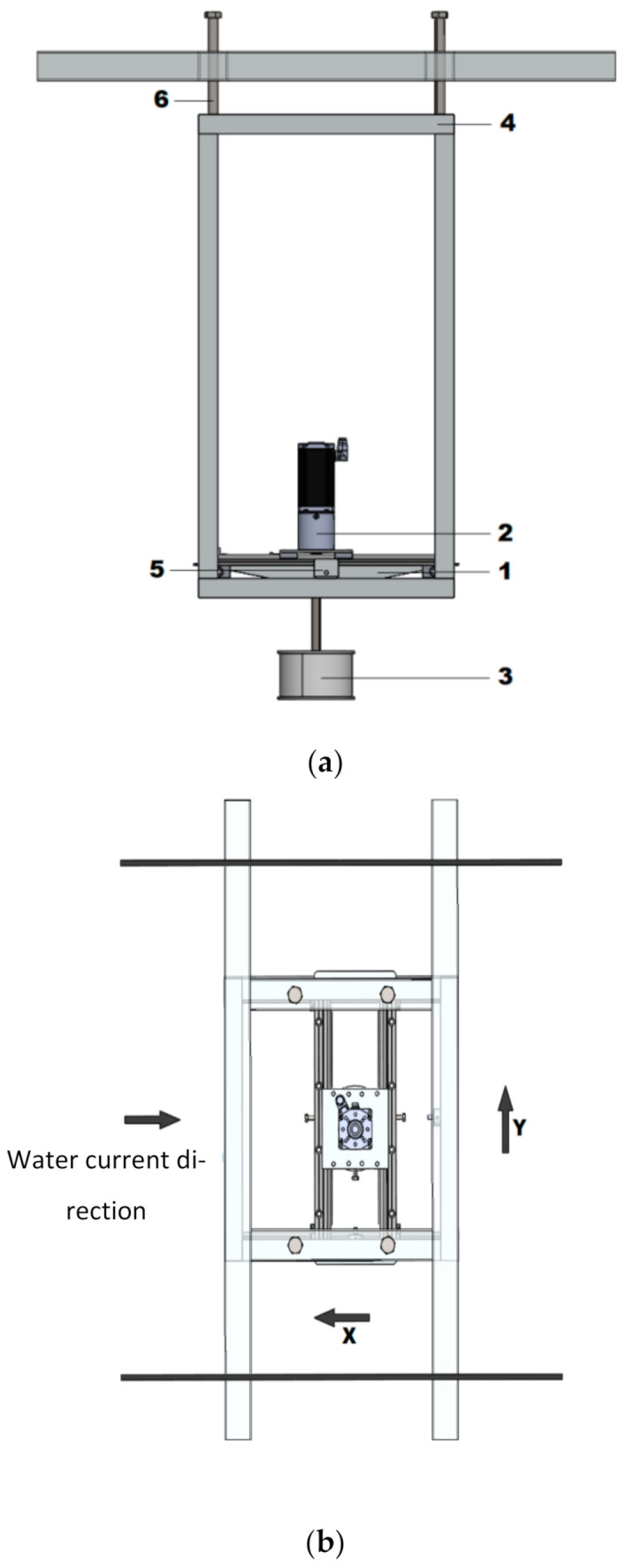
The force measuring part of the experimental test setup: (**a**) side view: 1—bearing-mounted platform, 2—drive system, 3—cylinder, 4—support frame, 5—force sensors, and 6—adjusting screw; (**b**) top view.

The force measuring part of the experimental test setup is placed on the supporting frame made of aluminum flat bars and aluminum profiles (Figure 3).

The construction of the frame allows for changing the immersion of the tested cylinder with adjusting screws. The main elements of the force measuring experimental test setup are BLDC (BrushLess Direct-Current) drive motors and replaceable cylinders.

The BG75X50SI drive is integrated with a planetary gear with a 1:4 ratio to ensure the appropriate system dynamics at rotational speeds of the cylinder in the range up to 1000 rpm and torque up to 5 Nm.

There are thermal and overload protections implemented in the control system to protect the drive in the event of uncontrolled dynamic states. The drive is controlled by the operator console equipped with a seven-segment display of the rotational speed, potentiometer regulating the drive voltage level, and a two-position switch enabling the change of the stator rotation direction.

The tested cylindrical rotors were made of PA6 polyamide, which is easy for technological processing, with high mechanical strength, low moisture absorption, low roughness, and optimal rotating mass. Each rotor was equipped with rounded end plates.

The entire drive system along with the measuring devices has been integrated with a bearing-mounted platform free to move in two directions along x and y axes.

In different solutions of force measuring devices, the rotor drive was mounted on a three-component force measuring balance [16], or two force gauges rigidly fixed to the drive shaft at the top and bottom of the rotating part of the cylinder [13]. The free movement of the measuring section, relative to the drive shaft, allows in this solution to measure its displacement using the strain gauges on the force gauges [13].

The free surface effect in the known experimental setups is eliminated by the plate situated on the water surface [16] or the non-rotating upper part of the cylinder crossing the free surface [16].

**Figure 4:**
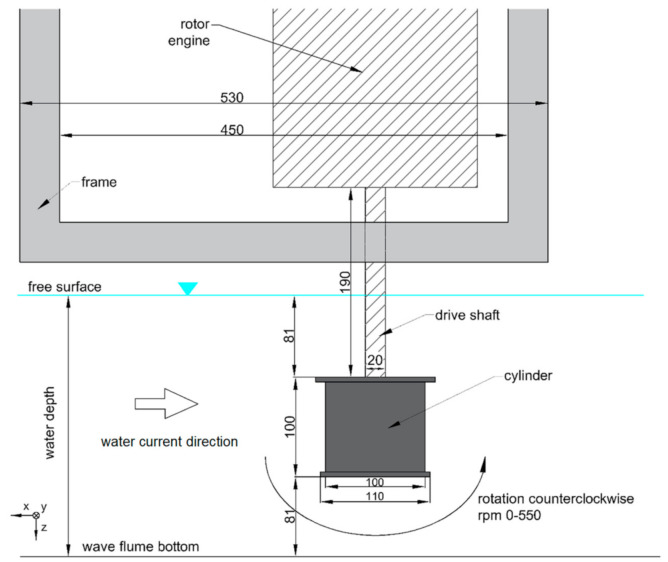
Location of the rotor, rotor drive, and supporting frame in the wave flume.

The scheme of the measurement area used in the presented experiment is presented in Figure 4. The rotating cylinder was located 25 m upstream from the water pump and its vertical position was in the center of the water column. The still water depth was equal to h = 0.262 m. The location of the rotor, rotor drive, and supporting frame in a wave flume and its dimensions are presented in Figure 4.

### 2.2. Measuring Sensors and Procedure

The main objective of experiments was to measure the lift force F_Y_ generated on the rotating cylinder in the presence of the stationary water current.

The water current velocity was recorded by two Acoustic Doppler Velocimeter probes located 5 m upstream and 5 m downstream of the rotating cylinder (Figure 2). The Nortek instruments measure the velocity of water by utilizing the Doppler Effect [19]. The ADV Vectrino, 3D Lab Probe measures the acoustic signal reflection from the seeding particles suspended in water with an operating acoustic frequency equal to 10 MHz, giving the water particles velocities with an error of ±0.001 m/s [19,20]. It requires a great amount of seeding material to provide its proper concentration in the whole volume of water.

The aim of those measurements was to confirm the stationary conditions of flow. Additionally, the water current velocities were determined indirectly by the flow rate of the pump.

The forces generated by the rotor were recorded using the KMM20 force sensors equipped with threaded fasteners capable to measure both the tension and compression. Their operation is based on a strain gauge bridge. The small size sensors with a wide measuring range of 200 N nominal rated forces and maintaining the accuracy class of 0.5 have proven themselves very well in this application. An additional advantage of the sensors was the built-in signal amplifiers, which significantly simplified data registration. The voltage readings of the transducer were incorporated into the DHI Filter Cabinet Type 155/IF controlled by the National Instruments DAQmx with the NI 6229 device and converted to forces. Linearity tolerance of the transducers was 0.25% of the reading.

The investigated cylinder diameters were as follows: D_1_ = 0.05 m, D_2_ = 0.10 m, and D_3_ = 0.15 m. The endplates diameters to the cylinders diameters ratios D_e_/D for all cylinders were equal to 1.1. The roughness coefficients of cylinders are as follows: Ra: 2.5 μm, 1.6 μm, and 4 μm respectively. The roughness of the cylinders was measured using a HOMMEL-ETAMIC W5 device equipped with a head for measurements on cylindrical surfaces. The measurement was performed five times in different places of each rotor. The mean value of the measurements was determined and used as the roughness coefficient in simulations.

For each cylinder, five current velocities were tested: 0.4 m/s, 0.5 m/s, 0.6 m/s, 0.73 m/s, and 0.82 m/s. The rotational speed of the cylinders was in the range 0–550 rpm with the step of 50 rpm (Table 2).

The cylinder rotated counterclockwise. The recording duration of a single measurement was 1 min for each rotational speed and 5-min gap between the subsequent rotor rotational speeds was made.

## 3. Results of Measurements

**Figure 5:**
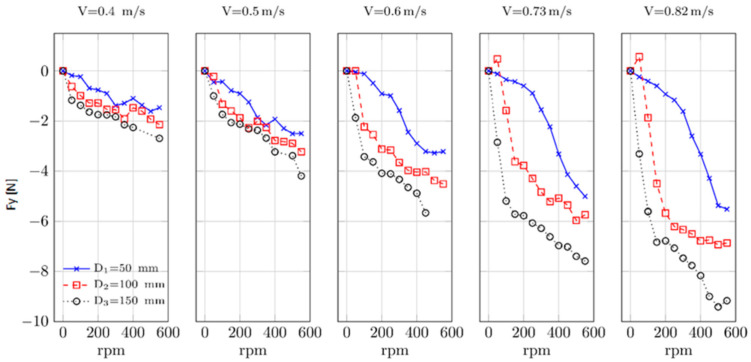
Lift force obtained from the measurements in the wave flume for different flow velocities and cylinder diameters.

The measured lift force F_Y_ in the adopted coordinate system (Figure 4) is presented in Figure 5. For low current velocities, the increase of absolute force values is constant through the whole rpm range, especially for the lowest considered cylinder diameter 0.05 m. The force is higher for higher cylinder diameters.

This increase of force for rotational speed up to 200–250 rpm follows the vortex shedding process, which creates the von Karman street in the wake of the cylinder [8]. Beyond certain rpm, this process deteriorates limiting further increase of the lift force. This effect can be seen in Figure 5 for the highest considered rotational speeds when the absolute value of the force seems to achieve its maximum. That means a further increase of the rpm would not lead to an increase of the force. The detailed investigation of this problem was carried out by Karabelas et al. [7].

The measurement error for the force was 1.2 N. The error related to non-linearity (% F.S.) was 0.25% of 200 N, hysteresis (% F.S.) was 0.25% of 200N, and the creep error 30 min (% F.S.) was 0.1% of 200 N.

The lift force coefficient C_L_ (1) related to α (2) was analyzed with respect to Re (3) for all the tested flow conditions and cylinder diameters used in the wave flume experiment.

(1) $$C_{L}=\frac{F_{Y}}{0.5{\rho V}^{2}\mathrm{LD}},$$

where: C_L_ is the lift coefficient, F_Y_ is the lift force obtained from the experiments, L is the height of the cylinder, and D is the cylinder diameter.

(2) $$\alpha =\frac{\mathrm{rD}}{2V},$$

where: α is the cylinder rotation rate, r is the angular velocity of the cylinder [rad/s], D is the cylinder diameter [m], and V is the free stream velocity [m/s].

(3) Re=VDν,

where: V is the free stream velocity [m/s], D is the cylinder diameter [m], and ν is the kinematic viscosity of water: ν = 10^−6^ m^2^/s.

Reynolds numbers for the tested flow velocities and cylinder diameters are presented in Table 3.

**Figure 6:**
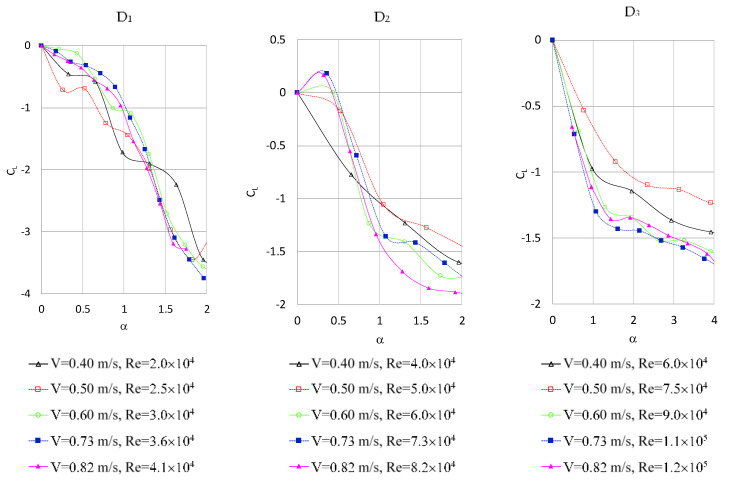
Variation of the lift coefficient with rotation rate for various free stream velocities and various cylinder diameters—experimental results.

The lift coefficients for α in the range 0–2 for the cylinders with D_1_ and D_2_ diameters and in the range 0–4 for the cylinder with D_3_ diameter are presented in Figure 6.

The comparative results are available for α no greater than 2 when the free surface effect at small rotational speeds can be neglected. The results obtained by different authors [7,10,15] are presented in Table 4.

In the present study, the measured values of C_L_ are in the ranges of values presented in the literature.

## 4. Numerical Computation of the Hydrodynamic Lift Force Generated by the Rotating Cylinder under Uniform Flow

### 4.1. Mesh Density and Turbulence Model

**Figure 7:**
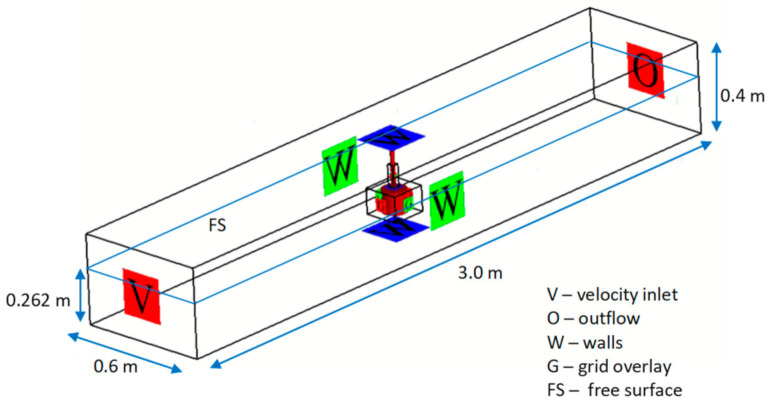
Boundary conditions for rotor-generated flow field simulation—computing domain with free surface level.

The numerical computations were carried out using FLOW-3D [21] program on the workstation with the Intel Xeon gold 6244, 3.60 GHz processor and RAM 96 GB volume. The computing domain with dimensions 3 m × 0.6 m × 0.4 m with free surface at 0.262 m was applied (Figure 7) to model the conditions of the experiment.

**Figure 8:**
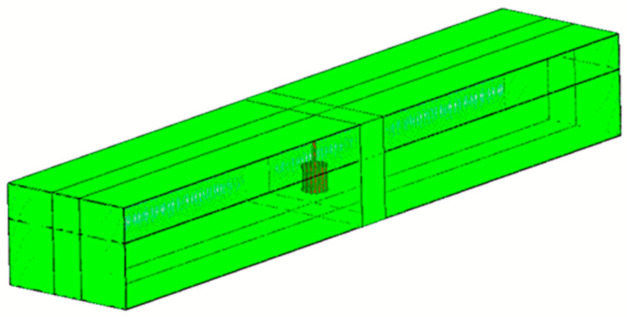
General view and the close-up of the rotor wall sector applied for the rotor simulation.

The general view and the close-up of the rotor wall sector applied for the rotor simulation is presented in Figure 8.

The following assumptions were used in CFD modeling. Computation was unsteady, with implicit pressure algorithm; “true” VOF (TruVOF) approaches were employed to capture the free surface position. The general moving object (GMO) model implemented in FLOW-3D was used for modeling rotational motion of the rotor. The rotational speed of the rotor started from 0 rpm and after 2 s achieved constant speed, in the range 50–500 rpm. The water flow velocity was in the rage presented in Table 2.

Four turbulence models were tested for the turbulence closure: standard k-ε model, renormalized Group (RNG) k-ε model, k-ω two-equation model, and large-eddy simulation (LES) model. The turbulent mixing length was dynamically computed using the structural rectangular mesh and the FAVOR™ technique (Fractional Area Volume Obstacle Representation) to discretize the rotor geometry.

The density of the mesh was selected primarily for mapping the shape of the rotor at the edges. The mesh cell size and number of cells for the tested meshes are presented in Table 5.

**Figure 9:**
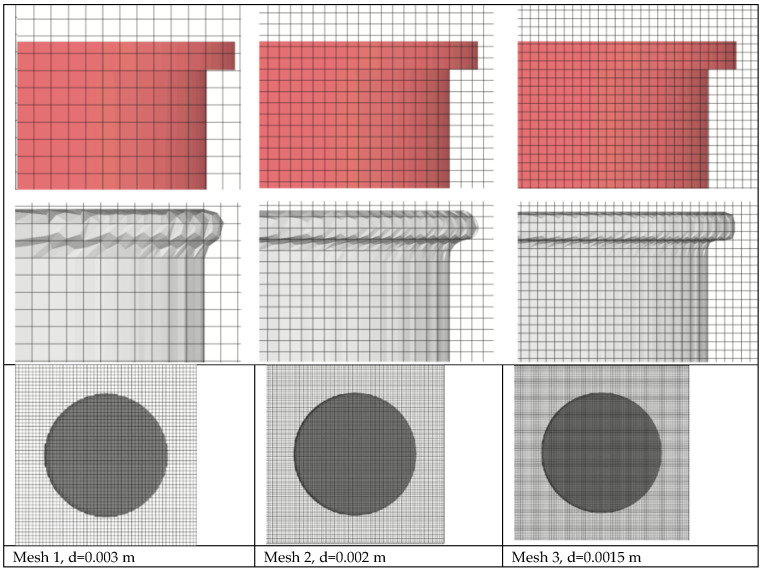
Structured mesh used in FLOW-3D and the FAVOR^TM^ technique—the original shape of the rotor and the shape of the object after FAVOR discretization technique for 3 mesh densities.

The FLOW-3D basic approach to gridding combines the advantages of rectangular grids with the deformed, body-fitted grids. The results of this method—the mesh density in the block near the rotor for the three mesh densities, which were chosen to test the influence of mesh resolution on calculation results—are presented in Figure 9.

The mesh technique used in Flow3D gives limited possibilities of boundary layer mesh near the wall and the FAVOR method does not precisely position wall locations within a cell. This is a disadvantage of this method because it causes high y+ values. The advantage of this type of meshing is the structural meshing in the whole domain, which improves the solution of free surface and reduces problems with complicated meshes during application of a moving object.

**Figure 10:**
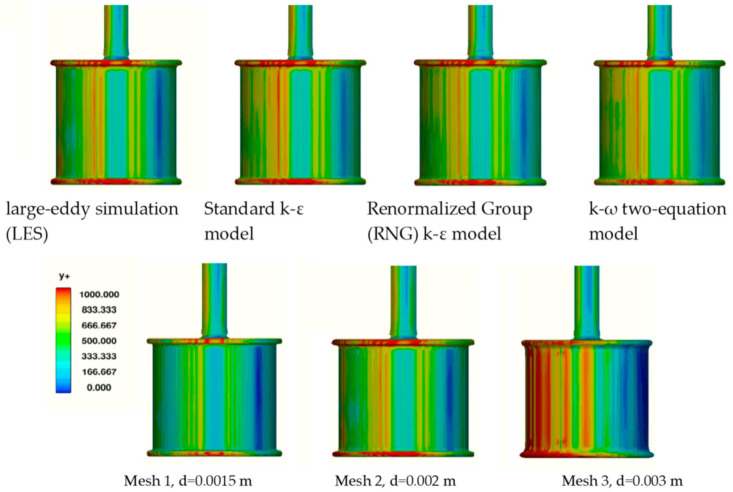
Parameter y+ for the studied turbulence models and meshes.

The mesh parameter y+ as a function of the cell size should be greater than 30, where the inner layer transitions smoothly into the log-law region and less than a value that depends on the Reynolds number of the flow and thickness of the boundary layer. Generally, 100 to 500 is accepted as a reasonable upper limit. The y+ parameter for the studied meshes is presented in Figure 10.

**Figure 11:**
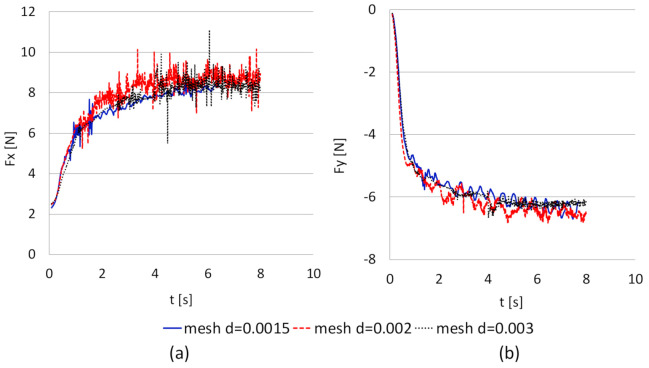
Results of numerical computations in time for the cylinder with D_2_ diameter at 500 rpm rotational speed and current speed V = 0.82 m/s using LES model in dependence of mesh density: (**a**) F_X_ and (**b**) F_Y_.

Despite the high values of y+, it was decided to compare the results, because increasing the mesh density in the entire area of the geometry significantly extends the computation time without changing the forces values. Also, for this reason, the turbulence models were tested at the same mesh densities. At first, the turbulence models were compared for Mesh 2, mapping the shape of the rotor, then the LES model, usually dedicated to model turbulent flows with rotating elements and giving the best convergence with the experiment, was selected (Table 6). The drag and lift forces F_X_ and F_Y_ obtained from CFD simulation in time t [s] using the LES model for the cylinder with D_2_ diameter at 500 rpm and V = 0.82 m/s in dependence of mesh density are presented in Figure 11.

The relative errors δF_X_ (4) and δF_Y_ (5) for the cylinder with a D_2_ diameter at 500 rpm, V = 0.82 m/s, mesh density 2.05 × 10^6^ (Mesh 2), and four selected turbulence models are presented in Table 6.

(4) $${\delta F}_{X}=\frac{F_{X\_\mathrm{EXP}}-{\;F}_{X\_\mathrm{CFD}}}{F_{X\_\mathrm{EXP}}},$$

(5) $${\delta F}_{Y}=\frac{F_{Y\_\mathrm{EXP}}-{\;F}_{Y\_\mathrm{CFD}}}{F_{Y\_\mathrm{EXP}}},$$

where: δF_X_, δF_Y_—relative errors of drag and lift forces, F_X_EXP_, F_Y_EXP_—measured drag and lift forces, and F_X_CFD_, F_Y_CFD_—computed drag and lift forces.

The relative errors δF_X_, δF_Y_ and time of computation in hours per 1 s of simulation [h/s] are presented in Table 7.

The differences between the experiment and CFD calculations may result from the small rotor aspect ratio, which causes turbulence in the area around the rotor. This turbulence caused strong vibrations during the experiment. On the other hand, the CFD simulation did not take into account rotor oscillation and did not model the entire flow around the rotor. The difficulties in the modeling of rotors have been reported by different authors [6]. Considering the calculation results and computer resources, the LES turbulence model and Mesh No. 2 with 2.05 × 10^6^ cells were selected.

### 4.2. Simulation of Rotor-Generated Flow Field

**Figure 12:**
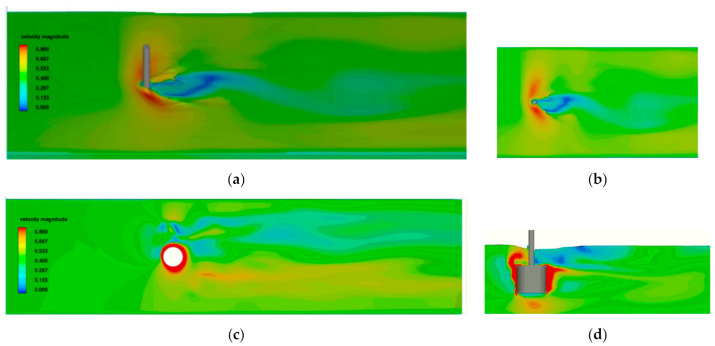
Results of 3D flow simulation for V = 0.40 m/s: (**a**) perspective view of velocity field on the free surface, (**b**) top view of velocity field on the free surface, (**c**) velocity field in the horizontal plane at half-length section of the rotor, and (**d**) velocity field in the rotor symmetry plane.

**Figure 13:**
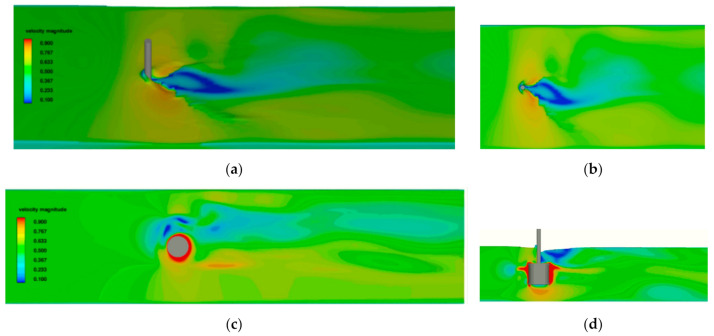
Results of 3D flow simulation for V = 0.50 m/s: (**a**) perspective view of velocity field on the free surface, (**b**) top view of velocity field on the free surface, (**c**) velocity field in the horizontal plane at half-length section of the rotor, and (**d**) velocity field in the rotor symmetry plane.

**Figure 14:**
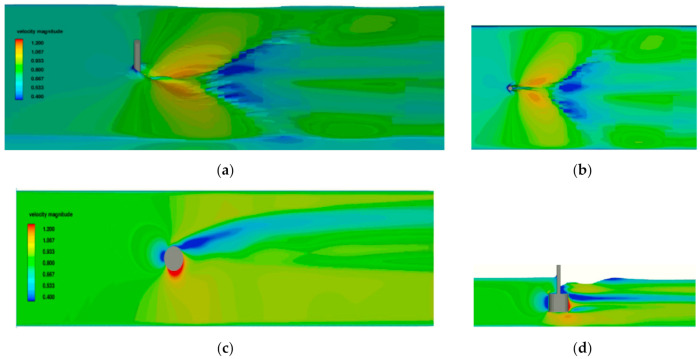
Results of 3D flow simulation for V = 0.82 m/s: (**a**) perspective view of velocity field on the free surface, (**b**) top view of velocity field on the free surface, (**c**) velocity field in the horizontal plane at half-length section of the rotor, and (**d**) velocity field in the rotor symmetry plane.

The examples of CFD simulations carried out to determine the flow velocity field and hydrodynamic force generated by the rotor with D_2_ diameter, aspect ratio D/L = 1 at 500 rpm, and different current velocities are presented in Figure 12, Figure 13 and Figure 14.

In Figure 12 and Figure 13, the area of higher velocity of 0.8 m/s is observed around the cylinder surface, and the small difference in distribution of pressure is the reason of small hydrodynamic lift and drag forces. The simulation showed that the unstable flow was the reason for strong vibrations during the experiment. In Figure 14, the significant difference in velocity on both sides of the rotor is the reason for pressure difference in the transverse direction and hydrodynamic lift force. The strong influence of free surface effect showed in Figure 14 causes the decrease in vertical distribution of the higher velocity area.

## 5. Discussion and Conclusions

**Figure 15:**
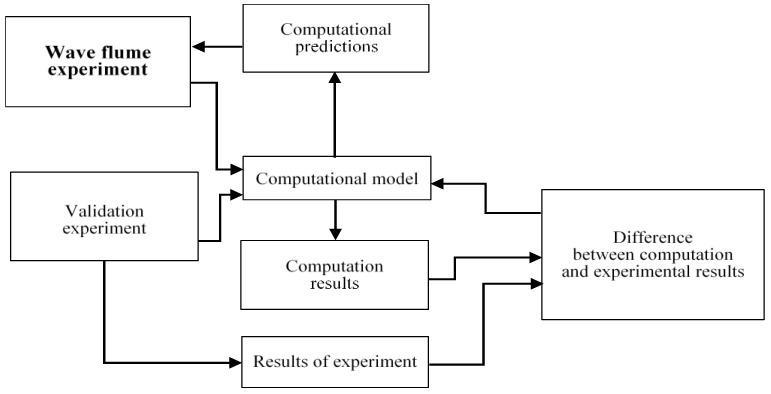
Flow chart of validation of the computational model against experimental results.

The model validation procedure is related to experiment verification—it involves graphical illustration and comparisons of the resultant flow field pattern and hydrodynamic force values for the assumed ranges of the influencing parameters. The validation process of the computational model is illustrated in Figure 15.

**Figure 16:**
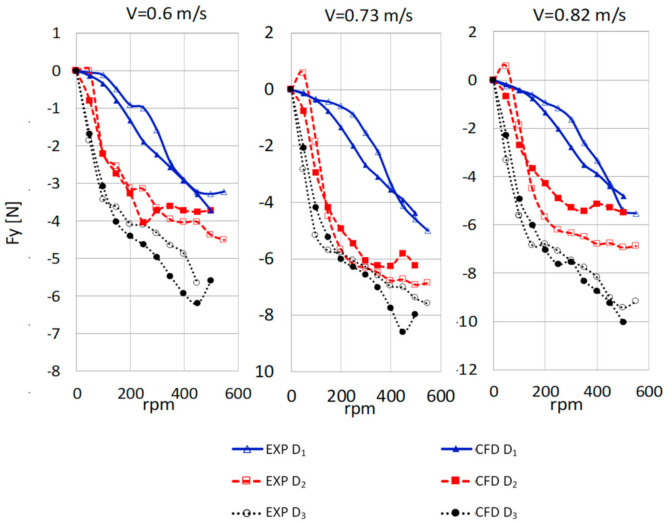
Measured (EXP) and computed (CFD) lift force values.

The measured and computed values of the lift force depending on the rotational speed for different rotor diameters and current velocities V = 0.6 m/s, 0.73 m/s, and 0.82 m/s are presented in Figure 16.

The conclusions are as follows:

1. If the computational results generally agree with the experimental data, the computational results will be regarded as validated and can be used for further predictions. The validation experiment allowed for determining the mesh density and turbulence model.
2. The computed values of the lift force for the rotor with diameter D_3_ at small current velocities 0.4 m/s and 0.5 m/s were different than the experimental values at the rotational speeds greater or equal to 200 rpm. The reason was the strong vibrations observed during the experiment as well as flow separation not modeled in the numerical simulation.
3. The opposite sign of the lift force was observed at small rpm for the rotor with a D_2_ diameter. This phenomena was not observed during simulations.
4. The presented experimental test setup was constructed to observe all phenomena including vibrations without causing damage to the drive, supporting structure, and measuring devices. Wang et al. [14] observed the vortex-induced vibrations increasing with the increase of the cylinder aspect ratio at the same value of α.
5. The smooth progress of the experiment was affected for α of about 4 with a possible damage of devices. In the present study, the strongest vibrations were observed for the cylinder of the largest diameter starting from α = 4.8.
6. The presented study described the method of reliable experimental prediction of the hydrodynamic force as a first part of further studies on the active control of rotor-generated flow [22] and bow steering system for river barges and vessels with expected good maneuverability, operated under current, wind, and waves [23].
7. The novelty of the paper is the investigation on the possibility of modeling the rotor-generated hydrodynamic force for the low aspect cylinders.
8. The main advantage of the method is a possibility of observing the rotor-generated flow field and hydrodynamic force, including phenomena related to the free surface effect and flow induced rotor vibrations. The proposed test set-up construction will be used in the parametric study of the hydrodynamic force, scale effect investigations, and validation of the CFD simulation model where big discrepancies were identified at low current velocity and big rotational speeds.

## Figures and Tables

**Table 1:** List of variables used in the paper.

Variable	Definition of Variable
C_L_ [-]	lift coefficient
d [m]	mesh cell dimension
D [m]	cylinder diameter
De [m]	cylinder endplate diameter
F_Y_ [N]	cylinder lift force
$F_{X\_\mathrm{CFD}}$ [N]	CFD computed drag forces
$F_{X\_\mathrm{EXP}}$ [N]	experimental measured drag forces
$F_{Y\_\mathrm{CFD}}$ [N]	CFD computed lift forces
$F_{Y\_\mathrm{EXP}}$ [N]	experimental measured lift forces
L [m]	cylinder height
r [rad/s]	cylinder angular velocity
Re [-]	Reynolds numbers
rpm [1/min]	cylinder rotational speed
V [m/s]	stream velocity
y+ [-]	mesh parameter—dimensionless wall distance
α [-]	cylinder rotation rate
${\delta F}_{X}$ [%]	relative errors of drag forces
${\delta F}_{Y}$ [%]	relative errors of lift forces
ν [m^2^/s]	kinematic viscosity

**Table 2:** Parameters ranges used during experiments.

**V [m/s]**	0.4	0.5	0.6	0.73	0.82
**rpm**	0–550	0–550	0–550	0–550	0–550
**D [m]**	0.05, 0.10, 0.15	0.05, 0.10, 0.15	0.05, 0.10, 0.15	0.05, 0.10, 0.15	0.05, 0.10, 0.15

**Table 3:** Reynolds numbers for all analyzed water flow conditions and cylinder diameter cases used in the wave flume experiments and numerical simulation.

V [m/s]	Re		
	D_1_ = 0.05 m	D_2_ = 0.1 m	D_3_ = 0.15 m
0.40	2 × 10^4^	4 × 10^4^	6 × 10^4^
0.50	2.5 × 10^4^	5 × 10^4^	7.5 × 10^4^
0.60	3 × 10^4^	6 × 10^4^	9 × 10^4^
0.73	3.6 × 10^4^	7.3 × 10^4^	1.1 × 10^5^
0.82	4.1 × 10^4^	8.2 × 10^4^	1.23 × 10^5^

**Table 4:** Lift coefficients.

Author	Re	α	C_L_
Tokumaru and Dimotakis [10]	3.8 × 10^3^–1.2 × 10^5^	1	0.8–1.2
		2	3–4
Mobini & Niazi [15]	1 × 10^4^	1.5	1.95
		2	2.75
Mobini & Niazi [15]	6 × 10^4^	1	0.75
Karabelas [7]	6 × 10^4^	1	1.15
Present study	2 × 10^4^–4.1 × 10^4^	1	1–1.8
	4 × 10^4^–8.2 × 10^4^		1–1.5
	6 × 10^4^–1.2 × 10^5^		0.6–1.3
	1 × 10^4^–4.1 × 10^4^	2	3–4
	4 × 10^4^–8.2 × 10^4^		1.4–1.8
	6 × 10^4^–1.2 × 10^5^		1–1.5

**Table 5:** Mesh cell size d and number of cells.

Mesh No.	d [m]	Number of Cells
1	0.0015	2.63 × 10^6^
2	0.002	2.05 × 10^6^
3	0.003	1.75 × 10^6^

**Table 6:** Influence of turbulence model on the relative errors δF_X_ and δF_Y_.

Turbulence Model	δF_X_ [%]	δF_Y_ [%]
Standard k-ε	3	18
k-ω	0	16
RNG k-ε	1	17
LES	2	10

**Table 7:** Influence of mesh density on the accuracy of computations and computing time.

Mesh No.	δF_X_ [%]	δF_Y_ [%]	Computing Time [h/s]
1	1	14	0.57
2	2	10	1.02
3	1	11	1.70
